# Drought-Adaptation Potential in *Fagus sylvatica*: Linking Moisture Availability with Genetic Diversity and Dendrochronology

**DOI:** 10.1371/journal.pone.0033636

**Published:** 2012-03-20

**Authors:** Andrea R. Pluess, Pascale Weber

**Affiliations:** 1 Ecosystem Management, Department of Environmental Systems Science, Swiss Federal Institute of Technology Zurich (ETH Zurich), Zurich, Switzerland; 2 Soil Functions and Soil Protection, Swiss Federal Institute for Forest, Snow and Landscape Research (WSL), Birmensdorf, Switzerland; Lakehead University, Canada

## Abstract

**Background:**

Microevolution is essential for species persistence especially under anticipated climate change scenarios. Species distribution projection models suggested that the dominant tree species of lowland forests in Switzerland, European beech (*Fagus sylvatica* L.), might disappear from most areas due to expected longer dry periods. However, if genotypes at the moisture boundary of the species climatic envelope are adapted to lower moisture availability, they can serve as seed source for the continuation of beech forests under changing climates.

**Methodology/Principal Findings:**

With an AFLP genome scan approach, we studied neutral and potentially adaptive genetic variation in *Fagus sylvatica* in three regions containing a dry and a mesic site each (*n*_ind._ = 241, *n*_markers_ = 517). We linked this dataset with dendrochronological growth measures and local moisture availabilities based on precipitation and soil characteristics. Genetic diversity decreased slightly at dry sites. Overall genetic differentiation was low (*F*_st_ = 0.028) and Bayesian cluster analysis grouped all populations together suggesting high (historical) gene flow. The Bayesian outlier analyses indicated 13 markers with three markers differing between all dry and mesic sites and the others between the contrasting sites within individual regions. A total of 41 markers, including seven outlier loci, changed their frequency with local moisture availability. Tree height and median basal growth increments were reduced at dry sites, but marker presence/absence was not related to dendrochronological characteristics.

**Conclusion and Their Significance:**

The outlier alleles and the makers with changing frequencies in relation to moisture availability indicate microevolutionary processes occurring within short geographic distances. The general genetic similarity among sites suggests that ‘preadaptive’ genes can easily spread across the landscape. Yet, due to the long live span of trees, fostering saplings originating from dry sites and grown within mesic sites might increase resistance of beech forests during the anticipated longer dry periods.

## Introduction

Climate change threatens to cause widespread modifications to forest composition and structure. Regional climate models for central Europe predict hot and dry summers and at the same time an increase in extreme precipitation events [1] causing most likely a spatial shift of species distributions due to species-specific climatic constraints. A geographically explicit modeling study for Switzerland indicated that the most common deciduous tree species, European beech (*Fagus sylvatica* L.), might be particularly vulnerable to the combination of the expected climatic conditions (niche model; [2]): With increasing temperatures and dryer summers, areas nowadays covered by beech forests are expected to shrink tremendously while areas at higher altitudes are expected to become suitable for this species. The predicted distribution is even more constraint if biotic interactions with co-occurring tree species were included in the model [3]. Yet, such models are restricted as they are calibrated by using a random sample of the populations studied [4] and thereby might undervalue the range limits. As *F. sylvatica* occurs in Switzerland across a multitude of environmental gradients including sites at its physiological limits, this species might be under divergent selection. If individuals at the dry distribution limits are adapted to lower moisture availability, *F. sylvatica* might contain the genetic variation for the continuation of beech forests under climate change even in the areas which are predicted to be devoid.

Selection driven genetic differences can be detected by Amplified Fragment Length Polymorphism (AFLP) genome scans, a useful method especially for non-model species which lack prior information on functional genes. Out of many gene fragments, those differentiated to a higher degree than the general background most likely are fragments which are part of genes under selection or are linked with such genes [5]. Indications for candidate markers can also be found if marker frequencies are associated with gradually changing environmental conditions [6]. This search for correlations with environmental variables is important for the understanding of which selective forces shape genetic differences [7].

AFLP patterns reflect a mixture of selection, drift and historical, demographic processes. To entangle the different processes, populations growing in similar environmental conditions but different regions need to be studied. Within region gene flow will diminish historical and demographic patterns leaving genetic imprints mainly driven by selection. If in different regions similar patterns between contrasting habitats are found, directional selection rather than drift most likely account for it. Gene flow might also reduce historical, demographic patterns beyond the regional scale. In genome scan approaches most fragments are expected to be neutral and thereby reveal mainly the historical, demographic component of the population structure as well as drift. Bayesian clustering analyses and isolation by distance tests indicate if populations from different regions are homogeneous and thereby can be treated as one group.

A number of studies on neutral genetic diversity and fitness indicate that diversity and fitness can be positively correlated [8]. In trees, dendrochronological characteristics such as growth increments and growth variation between subsequent years (i.e. sensitivity), can serve as fitness surrogate. If individuals at the boundary of the ecological envelope are adapted to the harsher environmental conditions, it can be expected that their growth is relatively constant even in harsher years. However, neutral genetic diversity might be depleted at the boundary of the ecological envelope [9] which might be reflected by high non-adaptive phenotypic plastic reaction to fluctuating environmental conditions.

The main aim of this study was to determine if divergent selection acted in *F. sylvatica* originating from nearby dry and mesic stands in three regions (Bärschwil, Neunkirch and Vetroz abbreviated with BAE, NEU and VET, respectively). Specifically, we investigated growth and AFLP variation in mature trees in stand-pairs containing one stand on a deep and the other on a shallow soil in ca. 500 m distance (i.e. mesic (m) and dry (d) stands called: BAEm, BAEd in the region BAE; NEUm, NEUd in the region NEU; VETm, VETd in the region VET). After comparing tree age, size and dendrochronolgical characteristics among sites, the analyses were threefold: First, general genetic diversity and differentiation patterns were investigated to test following hypotheses: (Ia) Genetic diversity is reduced at the species distribution limit. (Ib) Populations are of similar historical origin which is indicated by no difference among sites within regions or among regions. Second, with an outlier analyses mesic and dry sites within regions and across regions were compared to test following hypotheses: (II) Individuals are adapted to moisture availability indicated by markers which are more differentiated between mesic and dry habitats than expected under random processes. Third, marker frequencies were correlated with environmental and dendrochronological traits to test the hypotheses: (IIIa) Moisture availability determines the frequency of markers potentially under selection. (IIIb) The presence/absence of markers is related to growth characteristics, i.e. growth increments and sensitivity of growth increments, reflecting their adaptive character.

## Results

### Growth characteristics

At dry and mesic sites, trees under study were c. 120 years old (ANOVA: *P*_age_ = 0.29; *n* = 39 to 41 per site; Table 1). Tree height was up to double while diameter at breast height (DBH) tended to be higher at mesic compared with dry sites (*P*_height_ = 0.028, *P*_DBH_ = 0.076; for average values see Table 1). Age, tree height and DBH differed among sites (*P*<0.001 for all traits). Growth characteristics (median basal increment, BAI; median tree ring width, TRW; growth sensitivity, SEN; *n* = 9 to 11 per site) varied also among sites (*P*_BAI_<0.001, *P*_TRW_<0.001, *P*_SEN_ = 0.005) but did not differ between dry and mesic sites, despite BAI which tended to be larger at mesic sites (*P*_BAI_ = 0.080, *P*_TRW_ = 0.105, *P*_SEN_ = 0.512). These growth characteristics were similar at the two sites of the region VET. Excluding the data of this region from the analysis resulted into a threefold higher BAI and a 1.8 fold higher TRW at mesic sites, whereas SEN was similar between mesic and dry sites (*P*_BAI_ = 0.013, *P*_TRW_ = 0.006, *P*_SEN_ = 0.884). Mean deviation of TRW from the expected value (TRW_dev_) in the 20% driest years (determined via the drought index, DRI_June–Aug_, of the years 1930–2005) differed between dry and mesic sites with less negative values at dry sites whereas there was no difference in TRW_dev_ of the 20% wettest years (Dry years: −8.15±1.12 [1/100 mm] and −12.97±1.83 [1/100 mm] for dry and mesic sites, respectively; *t*-Test: *P*<0.03. Wet years: 7.21±1.06 [1/100 mm] and 9.42±1.68 [1/100 mm] for dry and mesic sites, respectively; *P* = 0.27).

**Table 1:** Genetic measures based on ten AFLP primer pairs, age and growth characteristics based on dendrochronological characteristics of *Fagus sylvatica* in three regions containing a dry and a mesic site each.

Group	Region	Site	*n*	%poly	*H*_e_	Age [y]	height [m]	DBH [cm]	BAI [cm^2^]	TRW [1/100 mm]	SEN
mesic	BAE	BAEm	41	85.69	0.233 (±0.008)	116.5 (±0.98)	34.8 (±0.72)	42.2 (±1.34)	23.3 (±3.07)	194.5 (±13.06)	0.224 (±0.011)
	NEU	NEUm	40	90.14	0.246 (±0.008)	113.1 (±4.07)	25.7 (±0.70)	47.8 (±2.00)	26.4 (±2.95)	184.9 (±15.52)	0.283 (±0.015)
	VET	VETm	41	89.56	0.235 (±0.008)	133.4 (±4.74)	24.4 (±0.72)	34.4 (±0.94)	10.3 (±1.15)	103.9 (±9.75)	0.235 (±0.008)
dry	BAE	BAEd	40	78.92	0.217 (±0.008)	129.0 (±2.11)	16.3 (±0.40)	28.6 (±0.78)	7.6 (±1.34)	109.1 (±13.03)	0.247 (±0.014)
	NEU	NEUd	39	82.01	0.226 (±0.008)	124.2 (±3.54)	18.2 (±0.42)	35.6 (±1.25)	9.6 (±1.48)	99.7 (±13.87)	0.248 (±0.019)
	VET	VETd	40	87.04	0.231 (±0.008)	134.9 (±5.90)	11.3 (±0.40)	26.5 (±1.08)	8.5 (±1.05)	94.3 (±7.11)	0.298 (±0.018)

*n*, number of samples; %poly, percentage of polymorphic AFLP loci; *H*_e_, expected heterozygosity; ± SE, standard error; DBH, mean stem diameter at 1.3 m height (cm); Age, mean age; TRW, median year ring width in 1957–2006; BAI, median basal increment in 1957–2006; SEN, average of the mean sensitivity of individual trees; TRW, BAI and SEN are based on ca. 10 trees per site.

### Genetic diversity

Of the 517 polymorphic AFLP-markers, on average 85.6% (SE = 1.79) were polymorphic within a given site (*n* = 39 to 41 per site; Table 1). Genetic diversity (*H*_e_) differed between sites in BAE and NEU with lower diversity at dry compared to the mesic sites (*t*-Test, Bonferroni corrected *P*-values: *P*_Bonf_<0.01 and *P*_Bonf_<0.001, respectively). *H*_e_ also differed between BAEd and NEUm, BAEd and VETm as well as VETd and NEUm (*P*_Bonf_<0.01 for all comparisons) while all other comparisons were non-significant. *H*_e_ was not related to average growth sensitivity of the stands or TRW but increased slightly with BAI (Spearmans's rank correlation: *P* = 0.50, *P* = 0.66 and *P* = 0.03, respectively).

Linkage between markers occurred in 0.12% of all pair-wise comparisons (*N* = 161 out of 133'386 comparisons). The outlier loci (see below) were not linked with each other.

### Genetic structure

The inbreeding estimate of each stand did not differ from zero and thus, Hardy-Weinberg equilibrium was assumed for the following analyses. Overall genetic differentiation was low (*F*_st_ = 0.028, *P*<0.001) and variation was neither explained by differences among regions nor between dry and mesic sites (*P*>0.05 for both tests). The majority of variation was explained within sites and 1.96% or 2.95% of the variation was explained between sites within regions or among sites in dry vs. mesic locations, respectively (*P*<0.001 for both tests).

Pair-wise site differences (*F*_st_) were 0.01–0.04 with 0.02, 0.01 and 0.03 within the regions BAU, NEU and VET, respectively (*P*_Bonf_<0.001 for all comparisons). Pair-wise *F*_st_ increased with increasing distances between sites (R^2^ = 0.16, *P* = 0.043) suggesting slight isolation by distance.

The Bayesian cluster analysis indicated no distinct grouping of the individuals: only one individual each from VETd and VETm clustered differently and the admixture analyses indicated that 16 of the 241 individuals originating from all but the BAEd site were admixed with less than 30% contribution to the smaller group.

### Genes related to selection

Using BayeScan [10], the Bayesian outlier analyses indicated eleven markers diverging among the three regions. These marker frequencies might be influenced by historic, demographic processes and were therefore excluded from the following analyses.

In summary, 13 markers showed a higher differentiation than expected under the null hypothesis of no differentiation between dry and mesic sites (Table 2). All outlier loci had positive α*_i_*-values indicating directional selection. Specifically, the analysis of all mesic vs. all dry sites detected three markers possibly under selection or linked to genes under selection. Comparisons of the two sites within BAE, NEU and VET indicated four, two and six markers, respectively, with a higher differentiation between the dry and mesic site than expected under the null model (Table 2). One marker (227_AGG_CAT) indicated differentiation among all dry and all mesic sites as well as between the two sites at VET and BAE but for the latter with a posteriory probability slightly below the threshold value.

**Table 2:** AFLP outlier analyses of *Fagus sylvatica* between dry and mesic sites across all regions as well as within individual regions.

				Marker frequency (*n*_indi_ = 241)					Marker frequency (*n*_indi_ = 60)				
Comparison	Marker	*F*st	Posteriory probability	dry		mesic			dry		mesic		
				mean	SE	mean	SE		mean	SE	mean	SE	
dry vs. mesic	227_AGG_CAT_Fs#	0.030	0.954	0.093	0.052	0.319	0.061	↑↑↑	0.008	0.008	0.049	0.024	↑↑↑
	124_ACT_CTA_Fs	0.028	0.921	0.277	0.051	0.548	0.07	↑↑↑	0.075	0.014	0.131	0.021	= ↑↑
	203_AAG_CTC_Fs	0.026	0.793	0	0	0.074	0.038	↑↑↑	0	0	0.025	0.001	↑↑↑
BAEd vs. BAEm	75_ACT_CTA_Fs	0.050	0.936	0.699	0.112	0.819	0.042	↑ = =	0.101	0.03	0.172	0.014	↑↑↑
	297_AAC_CAA_Fs	0.049	0.923	0.110	0.038	0.278	0.128	↑↑↑	0.008	0.008	0.033	0.021	↑↑ =
	232_AAG_CTC_Fs	0.042	0.824	0.673	0.075	0.860	0.023	↑ = ↑	0.151	0.015	0.197	0.014	↑↑↑
	227_AGG_CAT_Fs#	0.037	0.772°										
NEUd vs. NEUm	426_ACC_CAC_Fs	0.050	0.856	0.875	0.101	0.737	0.068	↓↓↑	0.202	0.039	0.140	0.031	↓↓↓
	72_AGG_CAT_Fs	0.043	0.790	0.033	0.022	0.148	0.03	↑↑↑	0	0	0.025	0.014	= ↑↑
VETd vs. VETm	204_ATG_CTA_Fs	0.060	0.928	0.227	0.040	0.385	0.117	↓↑↑	0.084	0.036	0.082	0.035	↑ = ↑
	227_AGG_CAT_Fs#	0.059	0.865										
	174_ATG_CAC_Fs	0.054	0.833	0.522	0.088	0.646	0.088	↑↓↑	0.110	0.031	0.155	0.015	↑↓↑
	178_AAG_CTC_Fs	0.056	0.827	0.797	0.091	0.730	0.098	= ↑↓	0.193	0.021	0.156	0.054	= ↑↓
	171_AAC_CAA_Fs	0.051	0.798	0.554	0.089	0.402	0.053	= = ↓	0.084	0.036	0.090	0.029	↑ = =
	321_ACC_CAC_Fs	0.060	0.795	0.899	0.058	0.778	0.044	= ↓↓	0.227	0.015	0.205	0.007	= ↓↓

Marker, the number indicates the length of the fragment, the letters indicate the selective base pairs of Mse1 and EcoR1;

# , markers significantly differentiated in more than one comparison;

° , posteriory probability slightly below the threshold of 0.79;

Marker frequency, average marker frequencies at the three dry and mesic sites; SE, standard error. ↑↓ = , indicate increasing, decreasing or similar frequency (i.e. <10% difference) of occurrence of the dominant maker in dry compared to mesic sites at BAE, NEU and VET, respectively; *n*_indi_ = 241, full dataset; *n*_indi_ = 60, data subset including individuals for which also dendrochonological measures are available.

The generalized linear model indicated a total of 41 markers which were related to one or two moisture measures: 16, 4 and 29 markers related positively or negatively to water holding capacity of the soil (AWC), field capacity (FC) and DRI_Sept–Aug_, respectively (*P*-values corrected with the False discovery rate approach: *P*_FDR_<0.05 for all tests; Table 3; see Table 4 for site specific AWC, FC and DRI_Sept–Aug_ estimates). A total of seven markers already identified by the outlier analyses did also change frequency gradually with the moisture availability. Eight of the 41 markers were significantly related to two moisture measures.

**Table 3:** AFLP markers of *Fagus sylvatica* which significantly related to AWC, FC or DRI_Sept–Aug_.

		AWC			FC			DRI_Sept–Aug_		
BayeScan result	Sample_Names	estimate	*P*	*P*_FDR_	estimate	*P*	*P*_FDR_	estimate	*P*	*P*_FDR_
dry vs. mesic	227_AGG_CAT_Fs	2.97E-02	1.93E-03	*	2.54E-02	2.60E-04	*			
dry vs. mesic	124_ACT_CTA_Fs	3.64E-02	1.78E-05	**				2.67E-03	2.17E-03	*
BAEd vs. BAEm	75_ACT_CTA_Fs				1.49E-02	1.54E-04	*			
BAEd vs. BAEm	232_AAG_CTC_Fs	3.87E-02	3.14E-04	*	1.61E-02	5.92E-05	*			
VETd vs. VETm	204_ATG_CTA_Fs	2.92E-02	7.15E-04	*						
VETd vs. VETm	174_ATG_CAC_Fs	2.95E-02	5.25E-04	*						
VETd vs. VETm	178_AAG_CTC_Fs	−3.04E-02	9.99E-04	*						
	203_ACT_CTA_Fs							3.59E-03	2.21E-03	*
	119_AAC_CTT_Fs							−3.74E-03	4.36E-04	**
	279_AAC_CTT_Fs							−6.29E-03	4.29E-04	**
	301_AAC_CTT_Fs							−3.29E-03	3.56E-04	**
	324_AAC_CTT_Fs							2.73E-03	1.57E-03	*
	357_AAC_CTT_Fs							−3.88E-03	4.60E-03	*
	117_ATG_CAC_Fs	−6.35E-02	1.30E-03	*						
	165_ATG_CAC_Fs							−3.42E-03	1.67E-03	*
	263_ATG_CAC_Fs	−3.82E-02	9.70E-06	**				−2.69E-03	1.71E-03	*
	113_ACA_CAA_Fs	−4.17E-02	1.22E-05	**				−2.51E-03	5.27E-03	*
	138_ACA_CAA_Fs	3.11E-02	2.33E-03	*				3.22E-03	5.82E-03	*
	142_ACA_CAA_Fs	−3.34E-02	6.53E-04	*				−4.05E-03	3.96E-04	**
	485_ACA_CAA_Fs							3.26E-03	4.49E-03	*
	88_AAC_CAA_Fs							2.62E-03	5.55E-03	*
	110_AAC_CAA_Fs							−2.59E-03	2.53E-03	*
	134_AAC_CAA_Fs							2.78E-03	3.19E-03	*
	163_AAC_CAA_Fs							−2.73E-03	4.59E-03	*
	164_AAC_CAA_Fs							2.59E-03	2.55E-03	*
	227_AAC_CAA_Fs							3.52E-03	1.35E-04	**
	270_AAC_CAA_Fs	−2.83E-02	1.31E-03	*						
	289_AAC_CAA_Fs							−3.60E-03	3.63E-03	*
	317_AAC_CAA_Fs	4.42E-02	1.67E-04	*						
	93_AGG_CAT_Fs							3.43E-03	8.84E-05	**
	110_AGG_CAT_Fs							7.08E-03	9.29E-04	*
	53_AAG_CTC_Fs	2.88E-02	4.96E-04	*	1.74E-02	4.32E-05	*			
	80_AAG_CTC_Fs							6.19E-03	4.08E-03	*
	358_AAG_CTC_Fs							−7.00E-03	4.81E-04	**
	364_AAG_CTC_Fs							3.92E-03	3.38E-04	**
	66_ACC_CAC_Fs							4.38E-03	4.04E-03	*
	132_ACC_CAC_Fs							2.44E-03	4.34E-03	*
	136_ACC_CAC_Fs							3.03E-03	4.59E-04	**
	139_ACC_CAC_Fs	−2.78E-02	2.06E-03	*						
	368_ACC_CAC_Fs							3.89E-03	4.48E-04	**
	131_AGG_CTC_Fs	−2.86E-02	6.59E-04	*						

AWC, available water capacity; FC, field capacity; DRI_Sept–Aug_, drought index; *P*, *P*-value of logistic regression corrected for multiple testing with the False discovery approach;

* , *P*_FDR_<0.05;

** , *P*_FDR_<0.01;

**Table 4:** Locations and environmental characteristics of the mesic and dry *Fagus sylvatica* stands under study.

Group	Region	Site	Easting	Northing	Elevation [m a.s.l.]	Aspect	AWC	FC	Precip [mm]	T [°C]	DRI_June–Aug_	DRI_Sept–Aug_
mesic	BAE	BAEm	600'937	248'964	670	N	61	136	1206	7.77	100.20 (±18.67)	702 (±35.58)
	NEU	NEUm	682'114	282'296	570	N	48	134	1000	8.2	46.91 (±15.25)	517 (±33.37)
	VET	VETm	585'609	122'163	1200	SW	79	126	1164	5.66	129.24 (±14.47)	824 (±34.03)
dry	BAE	BAEd	601'803	249'023	700	S	26	40	1163	8.02	66 (±18.86)	596 (±35.50)
	NEU	NEUd	681'728	281'862	530	SW	46	127	1016	8.15	12 (±14.68)	437 (±32.05)
	VET	VETd	585'482	122'273	1280	SW	40	70	1199	5.39	144 (±15.04)	867 (±35.18)

Coordinates in meters according to the Swiss topographical maps (Bundesamt für Landestopografie, Wabern, Switzerland); AWC, Available water capacity; FC, Field capacity; Precip, average annual precipitation sum; T, Average annual temperature; DRI_June–Aug_, DRI_Sept–Aug_ (Precip minus Pot. Evapotranspiration ± SE), Drought index for Sept. of the previous year to Aug. of the focal year and for June to Aug., respectively.

### Genes related to environmental and dendroecological characteristics

For the sixty trees with known growth sensitivities, the genetic dataset reduced to 319 polymorphic markers. The markers which were determined as genes related to selection in the full dataset did also occur at different frequencies in the reduced data set and often, the frequency changes from mesic compared to dry stands were similar to those in the full data set (Table 2). However, the outlier analyses did not determine any marker in the reduced data set to be significantly differentiated. Likewise, none of the marker frequency changed in accordance to the AWC, FC or DRI_Sept–Aug_ gradients (*P*_FDR_>0.05 for all tests).

The marker presence/absence was not related to the growth characteristics BAI, TRW and SEN (*P*_FDR_>0.05 for all pair-wise comparisons). Likewise, markers were not related to TRW_dev_ or the factor dry/wet years (*P*_FDR_>0.05 for all pair-wise comparisons). Leaving VET out, due to higher DRI measures at the dry site in this region, gave similar results (details not shown).

## Discussion

The outlier analyses indicated that *F. sylvatica* stands on shallow and deep soils are under divergent selection. Besides the three makers which differed between all mesic and all dry sites, we found an additional ten markers which differed between sites within a respective region. Seven of them had similar changes in marker frequencies between dry and mesic sites in at least one other region (Table 2) indicating that they might be related to differential selection pressure despite their absence in the overall analyses. The other three outliers followed a more diverse pattern and might either be under selection pressure of an unknown environmental gradient or they might result from local historical processes within a given region. They might also be false positives, which, however, are expected to occur at a relatively low rate (<1%) in the method applied here [11]. With the correlative approach, many of the outlier loci were found to change frequencies with AWC, FC and/or DRI_Sept–Aug_, confirming the former grouping into dry and mesic sites. Moreover, another 34 markers were found to be under differential selection suggesting that moisture availability is indeed a selective force shaping population genetics.

### Genetic diversity at dry and mesic sites

The sites at the moisture boundary contained slightly lower levels of genetic diversity. The center-periphery hypothesis proposes lower diversity at the boundary of a species occurrence, a pattern which might be mainly linked to population size [9]. In our study system, species occurrence is continuous within a region with trees located at the moisture limits while others are located on deeper soils. Hence, similar effective population sizes can be assumed. The reduced diversity levels might therefore be caused by increased selection pressures not allowing every genotype to establish rather than by population size at the species boundary of occurrence.

### Genetic connectivity among stands

In agreement with the phylogenetic study on *F. sylvatica* by Magri et al. [12] we found no pronounced pattern among regions which suggests little to no genetic structure derived by historical processes including re-colonization after the last glaciation time. Oddou-Muratorio et al. [13] estimated that gene flow was up to 140 m in *F. sylvatica* without taking immigrating genes from beyond the study areas into account (areas of ca 1.7, ca. 6.8 and ca. 3 ha with observed adult tree densities of 50, 19 and 44 ha^−1^, respectively, were studied). These distances were similar for contemporary and historical gene flow. Overall they found a tendency for slightly fat tailed pollen and seed dispersal kernels suggesting a moderate potential for long-distance dispersal. In our study at regional scale, we found very low differentiations between sites which are 170–870 m apart. Likewise, Jump et al. [14] found low differentiation between *F. sylvatica* populations in ca. 2 km distance. Gene flow among sites seems to be extensive in *F. sylvatica* indicating that only strong selection pressures have the potential for an imprint in the genome.

### *In situ* size measures

The size measures indicated that growth of *F. sylvatica* is limited at the dry sites. Even though individuals were of similar age, trees were shorter and had thinner stems. The reduced growth might be a phenotypic plastic reaction to limited resources at the sites with shallower soils; these soils might have a lower amount of soluble and thereby plant-available nutrients due to their lower water holding capacities. Indeed, ion concentrations in roots of *F. sylvatica* seedlings were decreased in a drought treatment compared with well watered plants independent of the water availability at their seed origins ([15]: P; [16]: K, Mg, Mn and Zn). However, all provenances from wet habitats but only some from dry habitats were drought-sensitive in physiological parameters and organic compounds [17]. These results suggest that plants are less well provided by nutrients in drier conditions and that there was some physiological adaptation to the water availability of the provenance origin. Likewise, adaptation in specific root area was found in a drought experiment with *F. sylvatica* provenances from dry and wet sites [18]. This potential for adaptation to water availability is reflected in the observed outlier alleles reported here which indicated microveolutionary changes between mesic and dry stands. These results suggest that the reduced growth at dry sites might be a combination of plastic reaction as well as adaptive reaction to water availability.

### Linking genetics with dendrochronology measures

Even though stands in the drier sites were genetically less diverse and showed reduced growth, there was no correlation of neutral genetic diversity values and growth. Likewise, we found no correlation of the growth sensitivity measures and marker frequencies which might be explained by the genetic determination of the trait under study and/or by the sample size. First, growth characteristics are most probably polygenic traits [19] and thus, individual markers might reveal little about them. Moreover, Bone and Farres [20] calculated evolutionary rates in previously published studies on plant species under expected selection pressures (e.g. for copper tolerance) and found that physiological traits evolve more rapidly than morphological traits. Second, in each *F. sylvatica* stand the growth patterns were analyzed in a subset of an average of ten trees. This reduced dataset was probably statistically not powerful enough to detect links of growth traits with changing marker frequencies. In accordance, no outlier loci were found in the reduced dataset, even though the outlier loci found in the full dataset often changed in their frequencies in a similar manner as in this reduced dataset.

### Management implications

It was recently also asked by other authors, if marginal beech provenances are candidate ecotypes for a continuation of beech forests in the anticipated climatic condition. While Rose et al. [18] found adaptation to drought in a common garden experiment with seedlings originating from provenances which were more than 1000 km apart, we found genetic differentiation in relation to water availability in neighboring stands. Dispersal across large distances is thereby not needed for the spread of ‘preadapted’ genes in *F. sylvatica*. Sites at the drought distribution limit occur interspersed with mesic sites and we showed that the two site types are genetically well connected. Moreover, most of the gene fragments, which were related to water availability, also occurred at the mesic sites suggesting that local adaptation to drier conditions can also be achieved through allele frequency changes *in situ*. However, if the reduced growth of the genotypes primarily occurring at dry sites to date would be at least partially adaptive, individuals with those gene combinations might be competitively inferior to genotypes of mesic sites. To sow seeds from dry sites and foster their saplings in mesic sites might therefore add to the resistance of beech forests in Switzerland in a changing climate.

## Materials and Methods

### Ethics Statement

All necessary permits were obtained for the described field studies. The permits were issued by the forestry authorities at communal and cantonal level.

### Study species

European beech (*Fagus sylvatica* L.) is a diploid, monoecious, wind-pollinated, highly outcrossed [21], deciduous forest tree. Individuals mature at an age of 40–60 years [22] and form nutlets which are gravity and/or animal dispersed (e.g. squirrels, jays, nuthatches, [23]). Masting years occur irregularly and often in years following summer drought [24], [25]. *F. sylvatica* is highly shade tolerant and occupies a wide ecological niche, occurring on deep as well as shallow soils with reduced water retention capacities in the latter [26]. Given rapidly changing soil depths across short geographic distances, nearby *F. sylvatica* stands can experience large differences in soil moisture availability.

In Switzerland, *F. sylvatica* occurs predominantly in the colline to the subalpine forest zones (ca. 400 to 1600 m a.s.l.) in the Swiss plateau, Jura mountains and the foothills of the Alps [27]. In total, *F. sylvatica* covers 18.3% of the forested area and plays an important role in timber production with an annual proportion of timber of 61.4% of all broad leafed trees and 15.0% of all tree species [28]. Regeneration in Swiss forests is mainly natural [29].

### Study sites

In each of three geographic regions (BAE, NEU, VET), one stand pair with one site on shallow (dry: BAEd, NEUd, VETd) and the other on deep (mesic: BAEm, NEUm, VETm) grounds were sampled (Table 4). The average distance between the dry and mesic sites within a pair was 0.54 km (SE = 0.20) and is covered with a continuous forest. At mesic sites, *F. sylvatica* was the only tree species present, whereas at dry sites it co-occurred with oaks and pines. The selected stands are close-to-natural forests, where forest management has been ceased for many years. We assume that the trees sampled originate from natural regeneration because in the Swiss lowlands forest management in the second half of the 19^th^ century fostered artificial regeneration of coniferous species with a shift to broad-leaved species only in the first half of the 20^th^ century [30]. The three regions were in distances of 87.1 km (BAE-NEU), 127.8 km (BAE-VET) and 186.4 km (NEU-VET).

Site specific climatic variables for the period 1961–1990 (Table 4) revealed average temperatures of 6.01–8.52°C and annual rain fall of 1000–1206 mm based on measurements of nearby climate stations (www.meteoschweiz.admin.ch) and interpolated across altitude with Daymet [31]. The drought indices for one growing period (DRI_Sept–Aug_) and the summer months June to August (DRI_June–Aug_) were calculated as precipitation minus the potential evapotranspiration as described in Turc [32]. DRI_Sept–Aug_ and DRI_June–Aug_ ranged from 437–867 and 12–144, respectively, with smaller values at dry sites in BAE and NEU but not in VET. Local soil profiles revealed a water holding capacity (i.e. available water capacities, AWC) of 26–79 and a field capacity (FC) of 40–136 with lower values at dry sites. AWC and FC were assessed according to AG Bodenkunde [33]. At NEU, AWC and FC were only slightly reduced at dry compared with mesic sites but DRI_June–Aug_ differed nearly fourfold due to different evapotranspiration in the SW compared with the N expositions (i.e. NEUd vs. NEUm, Table 4). At VET, DRI_Sept–Aug_ and DRI_June–Aug_ increased slightly at the dry site compared with the mesic counterpart owing to a higher annual precipitation (+35 mm). However also in VET, due to the soil depth and texture, the dry site is indeed drier than the mesic site.

### Sampling design and growth assessment

At each site, 39 to 41 dominant trees were selected and mapped with a triangular method including two reference GPS-points (total *n* = 241). Tree height and diameter at breast height (DBH) was measured and leaf samples were taken. From each tree a core was taken at 80–100 cm stem height to estimate the approximate tree age based on growth ring counts with a bias of plus ten years. From nine to eleven trees per site, one additional core was taken and the two cores were used to estimate three growth characteristics for the years 1957–2006: TRW, median tree ring width [1/100 mm]; BAI, median basal growth increment [cm^2^] and SEN, growth sensitivity measures, i.e. variation in growth between two consecutive years. Moreover, we calculated the deviation of TRW (hereafter called TRW_dev_) from the value expected given by a linear regression of individual growth across the years 1930 to 2006. We then averaged these TRW_dev_ across the 20% driest and 20% wettest years of the period 1930–2006 based on the DRI_June–Aug_ measures.

### AFLP genotyping

Total DNA was extracted from silica-dried leaf tissue following the protocol of the DNeasy 96 Plant Kit (Qiagen, Inc.). The AFLP fingerprinting was adapted from Vos et al. [34] using the restriction enzymes EcoRI and MseI and ten primer-pairs with three selective base pairs per primer (EcoR1-Mse1: ACT-CTA, AAC-CTT, ATG-CTA, ATG-CAC, ACA-CAA, AAC-CAA, AGG-CAT, AAG-CTC, ACC-CAC, AGG-CTC; see Materials and Methods S1 for a detailed description of the protocol).

Fragments were separated on an ABI 3730xl DNA Analyser (Applied Biosystems), fragment lengths and peak heights were scored automatically with the Genemapper software v.4.0 ™ (Applied Biosystems) and were extensively manually revised. The raw data (*n* = 857 markers, 50 to 500 bp long) was further processed with AFLPScore [35]. Locus and phenotype scoring thresholds were determined based on one to four repeats of 26–35 individuals per primer-pair (27.63%, SE = 0.37 repeats per primer-pair). Depending on the primer-pair, the locus selection threshold was 120–800 rfu (median = 350 rfu) and the mismatch error rate was 0.97%–3.5% (mean = 1.98%, SD = 0.31). The final dataset contained the presence/absence information of 517 markers which occurred in more than two individuals and had a frequency smaller than 1–3/*n* [36].

For the subsequent analyses, markers of the same size were assumed to be homologous. Linkage disequilibrium (LD) among all pair-wise marker comparisons was tested with Fisher's exact test on contingency tables and followed by the false discovery approach to account for multiple testing (FDR: [37]; FDR level set to 5%). LD and FDR were calculated in R [38].

### Data analysis

#### Growth assessment

To test if tree age, diameter, height and growth characteristics (TRW, BAI and SEN) differ between dry and mesic sites, hierarchical ANOVAs were calculated. Sites were nested in the moisture availability class (i.e. dry, mesic).

#### Genetic diversity, differentiation and clustering

To test the assumption of Hardy-Weinberg equilibrium (HWE), inbreeding estimates for each site were calculated based on individual's inbreeding coefficient *f*_AFLP_ (an analogue to *F*_is_) using FAFLPcalc [39]. The overall *f*_AFLP_ was low (*f*_AFLP_ = 0.05, SE = 0.016) and values per site did not differ from zero (average values: −0.009–0.111, Bonferroni corrected 95%- confidence intervals of each value included zero). We therefore run all following analyses under the assumption of HWE.

Standard genetic diversity measures, i.e. percentage of polymorphic loci (%poly) and expected heterozygosity (*H*_e_) averaged across all markers were calculated using Arlequin V.3.5.2.1 [40]. To test for pair-wise site differences of *H*_e_, paired *t*-Test across all markers were done. Sequential Bonferroni *P*-levels were used to account for multiple testing.

Genetic structure was assessed via global genetic differentiation (*F*_st_), hierarchical AMOVAs (among and within regions; among and within the moisture levels), site pair-wise differentiation (all three analyses done in Arlequin) followed by a Mantel test and finally, a Bayesian cluster analyses. In the Mantel test, *F*_st_/(1−*F*_st_) was regressed on ln(geographic distance) in GenAlEx [41]. For the cluster analyses, the geographic locations were used as a biologically relevant non-uniform prior distribution over space. With the ‘spatial clustering of individuals’ approach (BAPS 5.1, [42]), the numbers of distinct clusters (K) were estimated. Values from 1 to 12 were entered 10 times each in the optimization algorithm. Subsequently the admixture coefficient for each individual was determined in the ‘admixture based on mixture clustering’ approach with a minimum reference population size of two individuals, 99'999 iterations to estimate the admixture coefficients for the individuals and 4'999 iterations of 999 reference individuals. If individuals across all sites are genetically similar, the analyses for genes related to selection can be done without special measures to correct for historical effects.

#### Genes related to selection

Markers under selection were determined using the hierarchical Bayesian method BayeScan [10]. This approach was chosen above others because true selective markers were found with less false positive counts in a recent method comparison [11]. Moreover, it estimates *F*_st_ not only per loci but also specific to each population allowing for population-specific demographic histories and different levels of genetic drift [10]. The logit value of *F*_st_ is decomposed into a locus specific effect (*α*_i_) shared by all populations and a population specific effect (*β*_j_) shared by all loci [43]. The posteriori distribution of α*_i_* indicates whether a locus is under directional (positive values) or balancing (negative values) selection. We present results of markers with a false-positive rate <5% which translates into a posterior probability >0.79 and a Bayes Factor >3, representing a ‘substantial’ evidence for selection [44]. BayeScan was run with the standard options with 5000 iterations and a thinning interval of 20 resulting in a total number of 100'000 iterations. First, markers differing among the three regions were assessed and excluded from all following analyses to diminish potential historical effects. Second, markers under selection between all mesic vs. dry stands were assessed as well as between the mesic and dry stand within each region. Per outlier analyses four impendent runs were done and outlier loci with consistent results are presented.

#### Genes related to environmental and dendroecological characteristics

The estimated humidity analogous AWC, FC and DRI_Sept–Aug_ indicate that the six sites occur along a humidity gradient. These three measures are related to each other (variance inflation factor >3, [45]), thus we tested each variable independently. We calculated binomial generalized linear models to determine AFLP-markers with changing frequencies along the humidity gradient using centered values for AWC, FC and DRI_Sept–Aug_. To determine if certain AFLP-markers are related to growth sensitivity (SEN) we used the binomial linear mixed models with the grouping level ‘site’. We run an additional binomial linear mixed model using the TRW_dev_ and the dry/wet year class as explanatory variables. All models were run for each AFLP-marker consecutively and an FDR approach was applied to account for multiple testing. Analyses were done in R.

## Supporting Information

Materials and Methods S1 **AFLP genotyping protocol containing details on DNA extraction, digestion and ligation reaction, pre-selective and selective PCR as well as fragment separation and marker selection procedure.**
(DOCX)
