# Supplementary material for: Drought-Adaptation Potential in Fagus sylvatica: Linking Moisture Availability with Genetic Diversity and Dendrochronology
Source: PLoS One. 2012 Mar 20;7(3):e33636. doi: 10.1371/journal.pone.0033636 (PMC3308988; doi:10.1371/journal.pone.0033636)
Supplement: Materials and Methods S1 — AFLP genotyping protocol containing details on DNA extraction, digestion and ligation reaction, pre-selective and selective PCR as well as fragment separation and marker selection procedure. (DOCX) [file pone.0033636.s001.docx]

**Drought-adaptation potential in *Fagus sylvatica*: linking moisture availability with genetic diversity and dendrochronology**

*Andrea R. Pluess and P. Weber*

**Materials and Methods S1**

***AFLP genotyping***

Total DNA was extracted out of 10–13 mg of silica-dried leaf tissue following the protocol of the DNeasy 96 Plant Kit (Qiagen, Inc.). The AFLP fingerprinting method was adapted from Vos et al. [[34](#_ENREF_33)]: First, about 100 ng DNA was digested with the low frequency (EcoRI, 0.05 U/µL) and high frequency restriction enzymes (MseI, 0.05 U/µL) in a 40 µl reaction mix containing 1 × NEBuffer#2, and 0.1 mg/ml BSA for 2 hour at 37 °C. Second, 14 µl of ligation mix containing 0.08 mg/ml BSA, 0.79 × NE Buffer#2, 3.97 mM ATP, 3.46 µl EcoRi Adaptor mix, 3.46 µl Mse-I Adaptor mix, and 1.43 U/µl T4 DNA Ligase was added (enzymes and buffers originate from BioConcept). The Adaptor Mix was previously mixed and heated for 5 min at 65 °C and cooled down in the PCR machine (closed lid) within 2 hours containing each 5 µM EcoR1 or 47.5 µM Mse-I top-and bottom adaptor and 0.5 × NEBuffer#2. The ligation incubated at room temperature for 3 hours, followed by incubation at 65 °C for 10 minutes to terminate ligation. Third, pre-selective PCR I with the primers EcoR1+A (1.5 µM) and Mse1+C (1.5 µM) was done in a 21 µl reaction mix including 2 µl restriction-ligation product, 1 × reaction buffer GoTaq-Flexi-Kit (Promega, Inc.), 0.36 mM dNTP mix (Promega), 2.5 mM MgCl_2_, and 0.025 U/µl GoTaq-Flexi-DNA-Polymerase (Promega). The PCR condition were as follows: 2 min at 72 °C, followed by 20 cycles of 20 sec at 94 °C, 30 sec at 56 °C, 2 min at 72 °C, with a final step at 60 °C for 30 min. Fourth, selective PCR II with 10 primer combinations (EcoR1-Mse1: ACT-CTA, AAC-CTT, ATG-CTA, ATG-CAC, ACA-CAA, AAC-CAA, AGG-CAT, AAG-CTC, ACC-CAC, AGG-CTC) was done in a 15 µl reaction mix including 3 µl 1:5 diluted PCR I product, 0.083 µM EcoRI+Axx and 0.25 µM Mse-I+Cxx primers, 2.68 mM MgCl_2_, and the same concentrations of the other reagents as PCR I. All PCR’s II were run with initial denaturation of 2 min at 94 °C, then 10 cycles with 20 sec at 94 °C, 30 sec at 66 °C with a decrease of 1 °/cycle, 2 min at 72 °C, following 22 cycles with an annealing temperature of 56 °C and a final prolongation of 30 min at 60 °C. The EcoRI primers were labeled with fluorescent dye (6 FAM, VIC, NED or PET; Applied Biosystems). All PCRs were carried out on a Bio-Rad dyad cycler (Peltier Thermal Cyclers, Bio-Rad Laboratories, Inc.). Fragments were separated on an ABI 3730xl DNA Analyser (Applied Biosystems) in a mixture of 3 µl of PCR II added to 10 µl of Formamide with 0.3 µl 500®LIZ size standard (Applied Biosystems) and after denaturation at 95 °C for 3 min. The primer combinations used, were chosen out of test runs of 27 selective Eco-MSE-I primer combinations with a subset of 8 duplicated individuals originating across all six populations. Primer pairs with highly reproducible and polymorphic pattern were chosen for the screening of all individuals.

A total of 26 – 35 individuals per primer-pairs were repeated one to four times starting from the restriction-ligation step. The duplicates were located in different sample batches than their original samples.
